# News and Miscellany

**Published:** 1903-03

**Authors:** 


### News and Miscellany.

IMPORTANT SANITARY MEASURES now pending in the Legislature:

By Dr. J. S. Miller, of Milam, in the House: A bill to prevent substitution on physicians' prescription under heavy penalty for violation of its provisions. Reported favorably by Committee on Public Health. Will be reached during March.

By Dr. Johnson, of Lee: An act requiring disinfection of railway coaches, sleeping cars, street cars and public conveyances, and providing a penalty for violation thereof. Reported favorably. This bill is very popular and will doubtless pass, as it should do. I do not see what kind of argument can be made against it. It is primarily in the interest of the public, who now realize the fact that sleeping cars are almost always more or less infected, especially by tubercle bacilli, for during winter thousands of consumptives are coming South; secondly, in the interest of railroad companies themselves, for disinfection will render their roads popular and will protect them from suits for damage by persons who acquire disease by traveling in infected coaches. One great road recently had to pay a $15,000 damage suit. The fact that a road had disinfected its coaches exactly as required by law and in accordance with the instructions of the State Health Officer, would enable any company to defeat any such suit.

THE COCAINE BILL, by Judge English, in the House: Reported favorably. It puts very severe restrictions around the sale of cocaine, morphine, chloral and opium, too severe, I think. It puts it absolutely out of the power of the unfortunate habitues to procure his or her accustomed drug even on prescription of a physician, "unless actually ill." This clause should be stricken out; it is inhuman.

THE PUBLIC HEALTH BILL having been reported favorably by the Senate committee, was taken up by special arrangement Tuesday morning, March 3rd. It was presented by Senator Harper of Brazos at the request of a majority of the State Medical Association's Committee on State Board of Health, by whom the bill was prepared. It enlarges the scope and power of the present health system of the State for the better protection of the public health by enabling the health officials to deal with other diseases than those now guarded against by quarantine, by instituting sanitary measures for their prevention; changes the name of the Quarantine

Department to Department of Public Health and Vital Statistics; creates a Bureau of Vital Statistics within said department, and provides for collecting, recording and publishing the vital statistics of the State, and for other purposes.

This bill should become a law. It is badly needed, and if enacted it will be the means of saving thousands of lives annually. It will accomplish all that it was hoped or expected to accomplish by a State Board of Health and by a less complicated and less expensive means. The legislators are, many of them, dead set against creating new salaried offices, and the signing members of the Association's committee satisfied themselves that the bill as drawn is the best that we can hope to get, and, indeed, in their opinion, it is the best form of legislation that can be asked for. It fills the requirements. The situation in Texas is peculiar, and demands that the head of the Public Health Department should have, as he now has with regard to quarantine, almost autocratic powers. The existing quarantine law could not be and would not be changed to conform to any other method of dealing with disease.

THE PHARMACY BILL, by Representative Love, of Dallas: Reported favorably in House. It amends the existing pharmacy law, creates a State Board of Pharmacy consisting of "five persons learned in the science and art of pharmacy, and defines the qualifications of a pharmacist, who must pass examination before this board. It defines the powers and duties of the board, and fixes penalties for violation of its provisions. This is all done in the interest of the public safety and for the protection of the better element of the drug profession. It should pass.

---

EXAMINATIONS of urine, sputum, blood, pathological specimens, etc., made at reasonable rates by New Orleans Clinical Laboratory, 124 Baronne Street, New Orleans. O. L. Pothier, M. D., Charity Hospital. I. I. Lemann, M. D., Secretary. J. B. Guthrie, M. D.

---

WANTED.—I want representatives everywhere to write names and addresses and to mail out advertising of my Brunswick Piano and one-minute piano player. No experience required. Good wages paid weekly for all or spare time. Send stamp for particulars. Cronkright Wholesale Piano Dealer, Pittsburg, Pa.

---

THE DEFUNCT MEDICAL ADVERTISING BUREAU OF NEW YORK, after some two or three years in liquidation through the courts, finally paid 9 cents on the dollar. After dividing my share with a law firm I got exactly $13.94 in payment of my bill of $223.41 (money the bureau had collected for me from good advertising patrons). Even that was a pick-up.

---

New Orleans Polyclinic.—Sixteenth annual session opens November 3, 1902, and closes May 3, 1903. Physicians will find the Polyclinic an excellent means for posting themselves upon modern progress in all branches of medicine and surgery. The specialties are fully taught, including laboratory work. For further information, address New Orleans Polyclinic, Postoffice Box 797, New Orleans, La.

---

State Medical Association of Texas.
OFFICE OF THE PRESIDENT.

Houston, February 26, 1903.

*For Dr. F. E. Daniel.*

℞ A trip to San Antonio,
April 26, 1903.

Sig.: Take part in the sessions, and enjoy the social functions.

S. C. Red, President.

Meeting, April 28th to May 1st, inclusive.

Referring to the above, you bet I'll be there, and so will the famous, favorite, flowery, brilliant, breezy, brave and delightful I. N. Love, of the *Medical Mirror.* We urge all to attend, and can pledge them a jolly good time. Dr. Red's "prescription" will do you good, and will cure indigestion and laziness.

---

How's this after eighteen years continuous advertising with the "Red Back"?

New York City, February 2, 1903.

*Texas Medical Journal, Austin, Texas.*

Gentlemen: It is simply a matter of form that we advise that we will continue as patrons of the Texas Medical Journal. It is entirely unnecessary to give you this assurance, because as long as the Texas Medical Journal lives, and S. & D. are in existence, they will be inseparable.

Extending to you our profoundest thanks for your innumerable courtesies, and wishing the best of luck to the editor of the Journal, we are,

Very truly yours,

Sharp & Dohme.

---

I want to call attention to the advertisement of the Matthewson Laboratory. They are making Chaparin. It is a product of *Cha-*

*parro Amargoso,* a Southwest Texas plant, and it is claimed to possess valuable curative properties in eczema and other skin diseases. Investigate the claim. My readers will recall the fact that the late Dr. R. T. Knox, of Gonzales, gave great prominence to this plant a few years ago as a remedy for dysentery. I am told that it is in general use by the Mexican peasantry and laborers as a cure for bowel troubles. Dr. L. H. Henley, of Marshall, Texas, has been making experiments with it as a cure for eczema. He can give you some pointers. *Chaparro* contains also salicylic acid, phenol and camphor.

---

DEATH OF DR. JAMES STUART WATSON, of Manor.—Dr. Watson was a valued member of the medical profession and of society. He was one of the charter members of the Austin District Medical Society, and his death is greatly deplored. We are indebted to Rev. W. H. Trainum for the following:

Dr. Watson was born in Monroe county, Ky., March 7, 1847, united with the Christian church when twelve, graduated from the medical department of the University of Louisville in the class of 1874. He married Miss Laura Ford, of Glascow, Ky., who, with three daughters, are left to mourn his loss. He moved to Burnet, Texas, in 1878, and to Manor in 1897. He had accepted a position as physician for the Kirby Lumber Company, and had been at his post only a week when the end came. He retired on the night of February 9th in good health, the next morning he was asleep—during the night he had gone. No signs of struggle were seen. It was heart disease, so pronounced by physicians a few years before. His father and grandfather both died young from the same disease. The doctor was a member of both the county and State medical associations.

---

INJUNCTION to prevent substitution for Fairchild's Essence of Pepsin. The supreme court of New York, on October 22, 1902, in the case of Fairchild Bros. & Foster vs. Jno. H. Eberhardt et al., decreed as follows:

"Adjudged that the defendants, their clerks, agents, servants and employes, be, and they hereby are, enjoined and restrained perpetually from selling or dispensing either at the drug store of the said defendants at No. 622 Third Avenue, in the City of New York, or elsewhere, any Essence of Pepsine or pharmaceutical preparation of any sort or kind whatsoever, not manufactured by the plaintiff, in imitation of, or in substitution for, Fairchild's Essence of Pepsine, whenever Fairchild's Essence of Pepsine is prescribed or asked for, and from representing by any word or action that any preparation sold by said defendants, not manufactured by the plaintiff, is Fairchild's Essence of Pepsine, together with Forty-two and 72/100 Dollars ($42.72) costs, and an extra allowance of Two Hundred and Fifty Dollars ($250).

"J. A. O'Gorman, J. S. C.
"Thos. L. Hamilton, Clerk."

"Thos. L. Hamilton, Clerk.
"M. J. D. (Seal.)

